# Serum profiles of methotrexate after its administration in children with acute lymphoblastic leukaemia.

**DOI:** 10.1038/bjc.1982.47

**Published:** 1982-02

**Authors:** C. R. Pinkerton, S. G. Welshman, J. M. Bridges


*Br. J. Cancer* (1982) **45**, 300

***Short Communication***

### SERUM PROFILES OF METHOTREXATE AFTER ITS ADMINISTRATION IN CHILDREN WITH ACUTE LYMPHOBLASTIC LEUKAEMIA

C. R. PINKERTON*, S. G. WELSHMAN† AND J. M. BRIDGES‡

*From the *Royal Belfast Hospital for Sick Children and the Nuffield Department of Child Health of the Queen's University of Belfast, the †Department of Clinical Chemistry of the Belfast City Hospital, and the ‡Department of Haematology of the Queen's University of Belfast*

Received 28 August 1981 Accepted 5 November 1981

METHOTREXATE (MTX) has for many years been used in the maintenance phase of therapy for acute lymphoblastic leukaemia (ALL), but the dose, route of administration and timing still vary between protocols. It is now possible, with accurate immunoassay techniques, to measure and compare serum MTX concentrations in children receiving treatment with various maintenance regimens. The oral and the i.m. routes are currently used in the Medical Research Council United Kingdom Acute Lymphoblastic Leukaemia (UKALL) trials and the i.v. route has been used in a number of American studies (Simone, 1974). MTX concentrations after different routes have been previously studied in adults (Freeman-Narrod *et al.*, 1975; Calvert *et al.*, 1977) but the results are not necessarily applicable to children, owing to differences in age, disease and the doses used. In the study reported here serum MTX profiles were compared after a standard dose was given to the same patients after each of 3 routes, oral, i.m. and i.v.

Six children were studied. All were being managed according to UKALL protocols; 3 received oral MTX routinely, 1 received i.m. therapy and 2 had completed maintenance therapy 2 and 36 months before this study. MTX (15 mg/m$^2$) was first given by the oral route with water after an overnight fast. Blood samples were taken from an indwelling venous cannula at 0, 20, 40, 60 min and 1·5, 2, 3 and 4 h after administration. On a subsequent occasion, usually within a month, the same dose was given as an i.m. injection into the buttock (parenteral solution, 25 mg/ml). Finally, MTX was given as an i.v. bolus, diluted to 5 ml with normal saline and injected over 30 sec. Blood samples were taken at 0, 10, 20, 40, 60 min and 2, 3 and 4 h after the i.v. dose. Specimens of serum were analysed by enzyme-linked immunoassay (EMIT MTX Assay, Silva, Maidenhead).

Serum profiles were compared in relation to the peak MTX concentration, its timing and the drug levels up to 4 h. The paired *t*-test was used for statistical analysis.

Results are shown in the Figure. In all cases the oral route produced lower peak concentrations than the i.m. route ($P < 0·001$) and levels were generally lower throughout the period studied. MTX was also more rapidly absorbed from the i.m. site, with significantly earlier peak concentrations ($P < 0·02$). Although the initial concentrations at 10 and 20 min were very high with the i.v. dose ($10^{-5}$–$10^{-4}$M), the rapid decline over the first 3 h produced no significant difference between oral and i.v. after 2 h. Moreover, in Cases 1–3

Correspondence to: Dr C. R. Pinkerton, Department of Child Health, Institute of Child Health, 30 Guilford Street, London WC1N 1EH.

FIGURE.—Serum methotrexate profiles in 6 children after oral (□), i.m. (○) and i.v. (△) administration of a standard MTX dose (15 mg/m²).

MTX concentrations were higher with the oral route.

In most cases the i.m. route produced more sustained concentrations, with higher levels at 3 and 4 h than either of the other routes. Because of the rapid absorption of the i.m. dose there was no significant difference between i.m. and i.v. concentrations at 1 h. In Case 5 the lower concentration at 4 h with the i.m. route appeared to be due to poorer i.m. absorption than in other cases, rather than particularly sustained levels with the i.v. route. In most cases, the rapid decline in concentration with the i.v. dose produced lower levels beyond 2 h than with the i.m. dose.

Although most drugs are better absorbed from an injection site, this is not necessarily the case, and some have been reported to reach higher, more consistent concentrations after oral than i.m. administration. (Curry, 1977; Scott & Hawksworth, 1981). With MTX, the i.v. route produced by far the highest peak concentrations but these were brief, and rapid distribution and excretion caused an early decline. Serum levels greater than $10^{-5}$M may be of value in relation to resistant blast cells in which the concentrations achieved with the oral and i.m. routes may be inadequate to overcome limitations due to poor intracellular transport or high dihydrofolate reductase binding requirements (Bertino *et al.*, 1962; Harrap, *et al.*, 1971). This might explain the improved response in some resistant cases using infusion schedules (Djerassi *et al.*, 1967). In most patients, however, such high concentrations are probably unnecessary, and routine i.v. therapy may be related to increased neurological toxicity (Aur *et al.*, 1978). The rapid fall in serum MTX after an i.v. dose has been previously described in adults with psoriasis (Noble *et al.*, 1975) and a variety of solid tumours (Freeman-Narrod *et al.*, 1975). Quite marked interpatient variation in the rate of distribution after an i.v. dose was evident in this study, and was similar to that reported in adults (Huffman *et al.*, 1973). This may have been due to differences in tissue- or serum protein-binding associated with very high drug concentrations. By contrast, the patterns of serum levels with the i.m. route were generally very consistent, and in all cases peaks were at least twice as high as those with the oral route. The slower decline in concentrations after an i.m. dose may have been due to continued uptake of drug from the injection site. This has been described in adults (Freeman-Narrod *et al.*, 1975; Calvert *et al.*, 1977) and furthermore it has been suggested that this is associated with a better therapeutic response in some solid tumours (Freeman-Narrod *et al.*, 1975).

In the interpretation of serum profiles after different routes of administration, it is important to consider the possibility that assay cross-reaction occurs between MTX and its metabolites. 4-Amino-4-deoxy$N^{10}$-methylpteroic acid (APA), for example, may result from bacterial action in the gut, and consequently influence oral profiles. This is, however, more important with high-dose schedules and prolonged studies, where metabolite concentrations are likely to be high.

Previous studies in children with ALL have demonstrated poor oral absorption in some cases. This is usually characterized by a slow rate of absorption and peak concentrations less than 0·5 $\mu$M (Kierney *et al.*, 1979; Pinkerton, 1980). As such poor absorption may contribute to therapeutic failure in such cases, might not the parenteral route be of value in these patients? It is clear from these data that the i.v. route has no particular advantages, but compared with the oral the i.m. route produces higher and earlier peak concentrations and a greater degree of absorption, as reflected in the higher concentrations up to 4 h. Another possible advantage might be increased patient compliance, as therapy would be given under supervision. This route is, however, both inconvenient and uncomfortable. To date there is no adequate clinical information to justify its routine use, but it may have a role where oral absorption is demonstrated to be poor.

A limitation shared by all 3 routes was the brevity for which serum concentrations were maintained above $10^{-7}$M. Studies *in vitro* have indicated that the maximum cytotoxicity is likely to occur where the extracellular drug concentration is maintained at $\sim 10^{-6}$M (Goldman, 1977). As such concentrations are achieved for only a few hours the therapeutic effectiveness of current regimens may not be optimal. Further studies are required to determine whether alternative schedules involving higher doses, possibly divided over 24 h, might produce advantageous serum profiles.

We are grateful to Mr H. Mackey and Mr M. McMaster of the Belfast City Hospital and to the haematology laboratory staff of the Belfast Hospital for Sick Children for technical assistance. C.R.P. was supported by a Royal Belfast Hospital for Sick Children clinical research fellowship.

### REFERENCES

AUR, R. J. A., SIMONE, J. V., VERSOZA, M. S. & 8 others (1978) Childhood acute lymphocytic leukaemia. *Cancer*, **42**, 2123.

BERTINO, J. R., DONOHUE, D. R., GABRIO, B. W. & 4 others (1962) Increased levels of dihydrofolate reductase in leukocytes of patients treated with amethopterin. *Nature*, **192**, 140.

CALVERT, A. H., BONDY, P. K. & HARRAP, K. R. (1977) Some observations on the human pharmacology of methotrexate. *Cancer Treat. Rep.*, **61**, 1647.

CURRY, S. H. (1977) *Drug Disposition and Pharmacokinetics*. Oxford: Blackwell. p. 99.

DJERASSI, I., FARBER, S., ABIR, E. & NEIKIRK, W. (1967) Continuous infusion of methotrexate in children with acute leukaemia. *Cancer*, **20**, 233.

FREEMAN-NARROD, M., GERSTLEY, B.J., ENGSTROM, P. F. & BORMSTEIN, R. S. (1975) Comparison of serum concentrations of methotrexate after various routes of administration. *Cancer*, **36**, 1619.

GOLDMAN, I. D. (1977) Effects of methotrexate on cellular metabolism: Some critical elements in the drug–cell interaction. *Cancer Treat. Rep.*, **61**, 549.

HARRAP, K. R., HILL, B. T., FURNESS, M. E. & HART, L. I. (1971) Sites of action of amethopterin: Intrinsic and acquired drug resistence. *Ann. N.Y. Acad. Sci.*, **186**, 312.

HUFFMAN, D. H., WAN, S. H., AZARNOFF, D. L. & HOOGSTRATEN, B. (1973) Pharmacokinetics of methotrexate. *Clin. Pharmacol. Ther.*, **14**, 572.

KEARNEY, P. L., LIGHT, P. A., PREECE, A. & MOTT, M. G. (1979) Unpredictable serum levels after oral methotrexate in children with acute lymphoblastic leukaemia. *Cancer. Chemother. Pharmacol.*, **3**, 117.

NOBLE, W. C., WHITE, P. M. & BAKER, H. (1975) Assay of therapeutic doses of methotrexate in body fluids of patients with psoriasis. *J. Invest. Dermatol.*, **64**, 69.

PINKERTON, C. R., WELSHMAN, S. G., DEMPSEY, S. I., BRIDGES, J. M., & GLASGOW, J. F. T. (1980) Absorption of methotrexate under standardized conditions in children with acute lymphoblastic leukaemia. *Br. J. Cancer*. **42**, 613.

SCOTT, A. K. & HAWKSWORTH, G. M. (1981) Clinical pharmacology: Drug absorption. *Br. med. J.*, **282**, 462.

SIMONE, J. (1974) Acute lymphocytic leukaemia in childhood. *Semin. Hematol.*, **11**, 25.


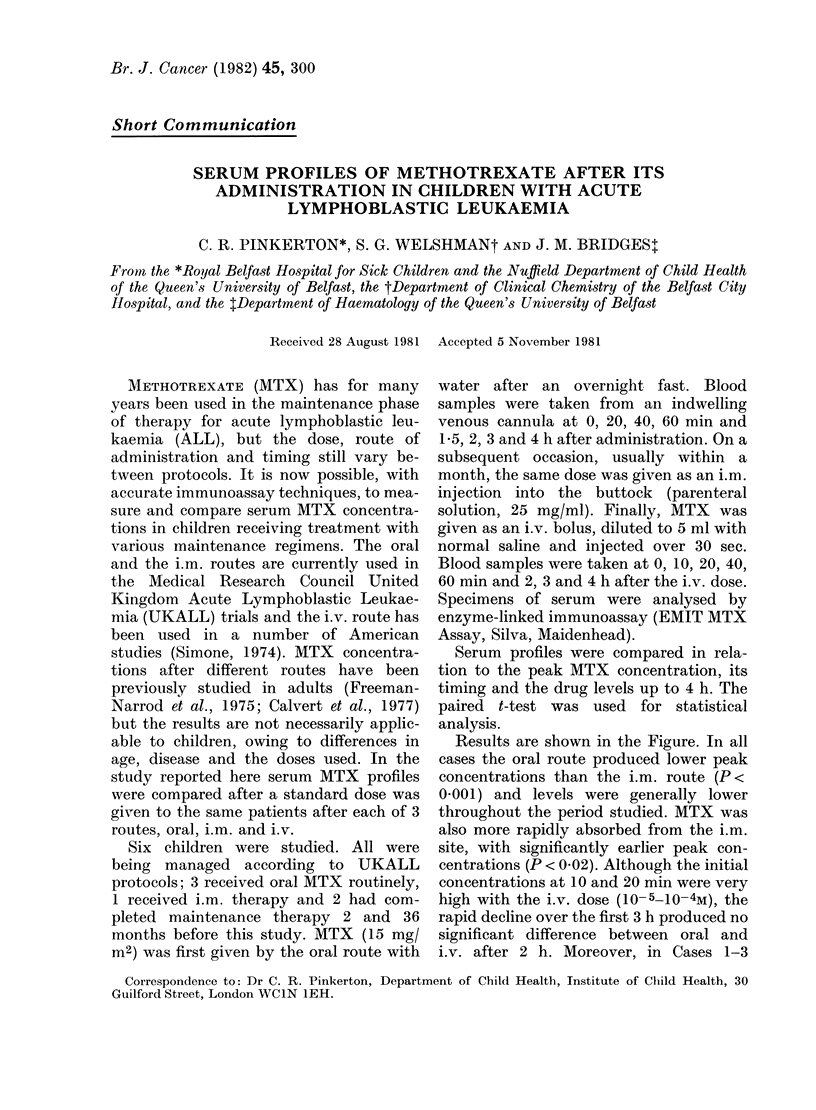

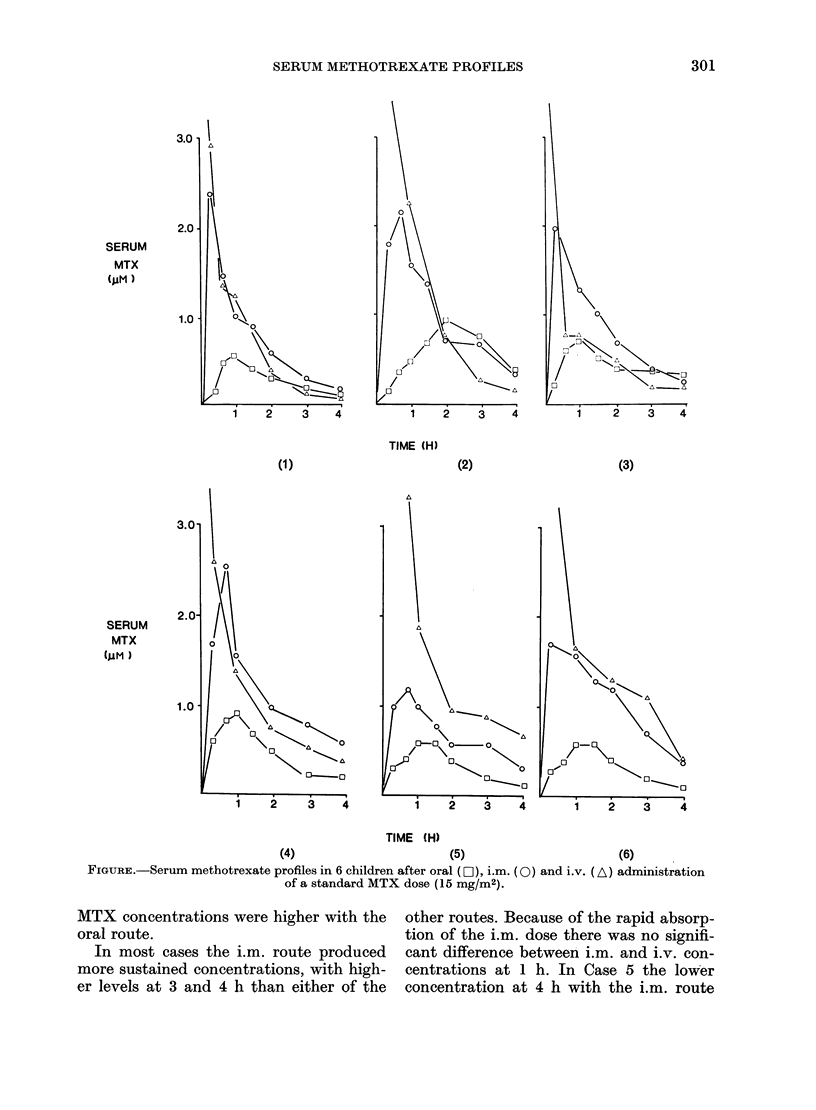

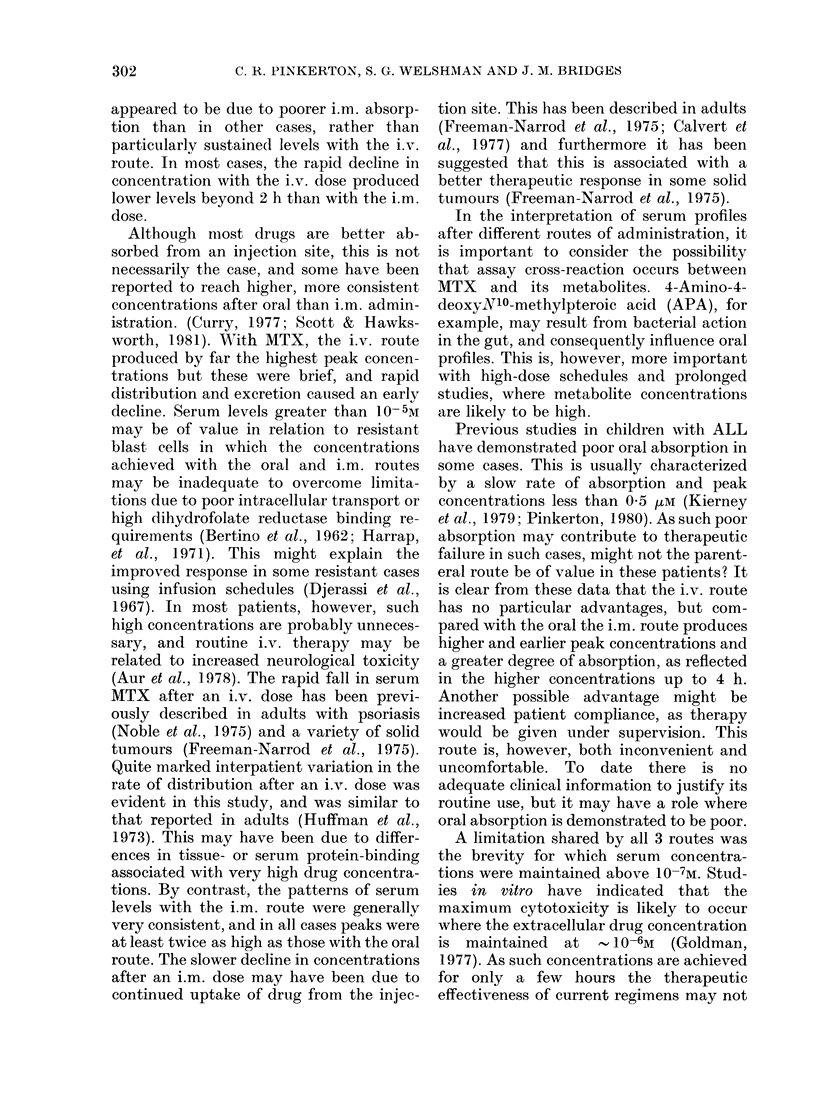

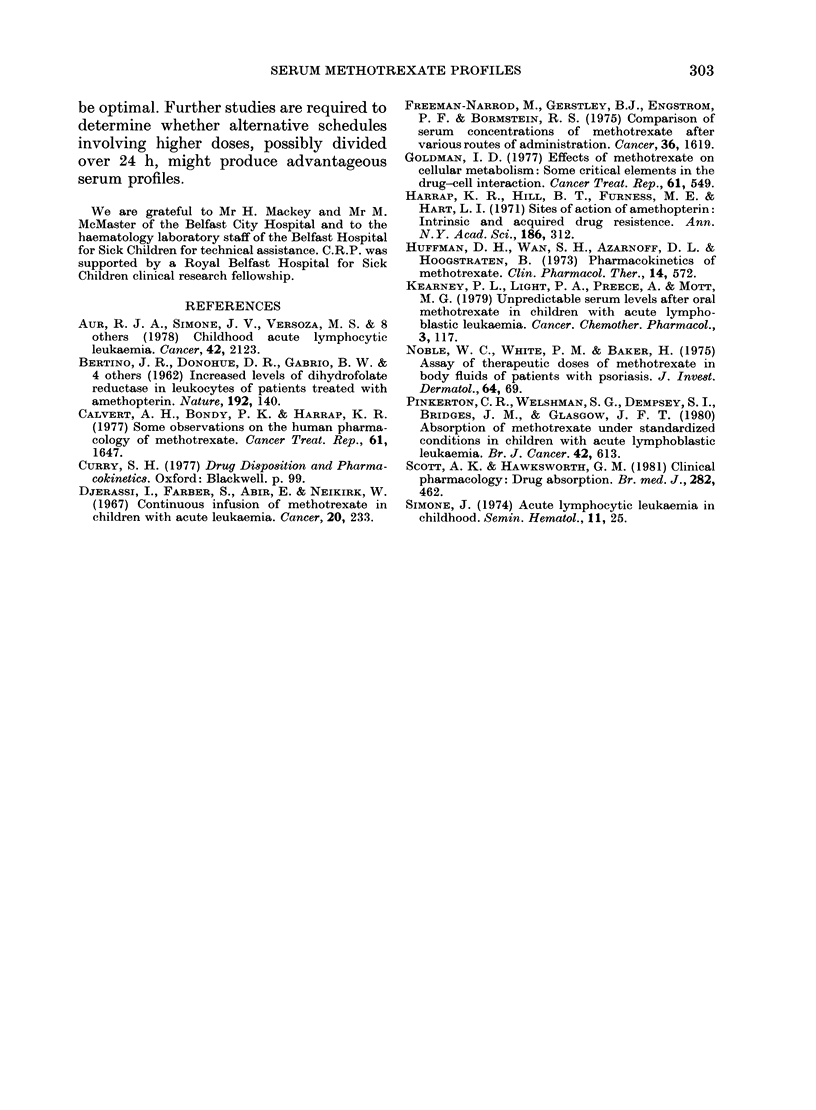


## References

[OCR_00276] BERTINO J. R., DONOHUE D. R., GABRIO B. W., SILBER R., ALENTY A., MEYER M., HUENNEKENS F. M. (1962). Increased level of dihydrofolic reductase in leucocytes of patients treated with amethopterin.. Nature.

[OCR_00282] Calvert A. H., Bondy P. K., Harrap K. R. (1977). Some observations on the human pharmacology of methotrexate.. Cancer Treat Rep.

[OCR_00292] Djerassi I., Farber S., Abir E., Neikirk W. (1967). Continuous infusion of methotrexate in children with acute leukemia.. Cancer.

[OCR_00297] Freeman-Narrod M., Gerstley B. J., Engstrom P. F., Bornstein R. S. (1975). Comparison of serum concentrations of methotrexate after various routes of administration.. Cancer.

[OCR_00302] Goldman I. D. (1977). Effects of methotrexate on cellular metabolism: some critical elements in the drug-cell interaction.. Cancer Treat Rep.

[OCR_00306] Harrap K. R., Hill B. T., Furness M. E., Hart L. I. (1971). Sites of action of amethopterin: intrinsic and acquired drug resistance.. Ann N Y Acad Sci.

[OCR_00312] Huffman D. H., Wan S. H., Azarnoff D. L., Hogstraten B. (1973). Pharmacokinetics of methotrexate.. Clin Pharmacol Ther.

[OCR_00317] Kearney P. J., Light P. A., Preece A., Mott M. G. (1979). Unpredictable serum levels after oral methotrexate in children with acute lymphoblastic leukaemia.. Cancer Chemother Pharmacol.

[OCR_00324] Noble W. C., White P. M., Baker H. (1975). Assay of therapeutic doses of methotrexate in body fluids of patients with psoriasis.. J Invest Dermatol.

[OCR_00330] Pinkerton C. R., Welshman S. G., Dempsey S. I., Bridges J. M., Glasgow J. F. (1980). Absorption of methotrexate under standardized conditions in children with acute lymphoblastic leukaemia.. Br J Cancer.

[OCR_00337] Scott A. K., Hawksworth G. M. (1981). Clinical pharmacology: drug absorption.. Br Med J (Clin Res Ed).

[OCR_00342] Simone J. (1974). Acute lymphocytic leukemia in childhood.. Semin Hematol.

